# Supplementary material for: The ACE2 Receptor from Common Vampire Bat (Desmodus rotundus) and Pallid Bat (Antrozous pallidus) Support Attachment and Limited Infection of SARS-CoV-2 Viruses in Cell Culture
Source: Viruses. 2025 Mar 31;17(4):507. doi: 10.3390/v17040507 (PMC12031370; doi:10.3390/v17040507)
Supplement: Supplementary file 1 [file viruses-17-00507-s001.zip › viruses-3499106-Figures S1-S3 and Table S1.docx]

**Table S1.** Protein identity matrix of ACE2 proteins. Pairwise identities of ACE2 proteins studied here are tabulated. Identity is shown in percentages. CVB and PB ACE2 are show in bold italics and bold respectively.

|  | **Human** | ***Vampire bat*** | **Pallid bat** | **Goat** | **Horse** | **Pig** | **Brazilian free-tailed bat** | **Chinese rufous horseshoe bat** | **Egyptian rousette** | **Greater Horseshoe bat** | **Pearson's horseshoe bat** | **Great roundleaf bat** | **Little brown bat** | **Chicken** |
| --- | --- | --- | --- | --- | --- | --- | --- | --- | --- | --- | --- | --- | --- | --- |
| **Human** |  | ***79.5*** | **79.2** | **81.6** | **86.8** | **81.4** | **80.1** | **80.6** | **79** | **81.2** | **81.4** | **80.5** | **79.3** | **65.6** |
| **Vampire bat** | **79.5** |  | **80.2** | **78** | **82.9** | **80** | **81.7** | **78.8** | **77.1** | **78.9** | **78.3** | **78.5** | **78.8** | **63.8** |
| **Pallid bat** | **79.2** | ***80.2*** |  | **78.6** | **82.8** | **79.1** | **83.5** | **79.5** | **79** | **80.7** | **80.2** | **80.8** | **88.3** | **62.9** |
| **Goat** | **81.6** | ***78*** | **78.6** |  | **85.2** | **87.7** | **80.6** | **80** | **79.1** | **80.2** | **80** | **78.9** | **78.3** | **65.4** |
| **Horse** | **86.8** | ***82.9*** | **82.8** | **85.2** |  | **87.1** | **83.6** | **84.6** | **82.9** | **85.5** | **85** | **84.6** | **82** | **66.5** |
| **Pig** | **81.4** | ***80*** | **79.1** | **87.7** | **87.1** |  | **81.5** | **80** | **80.2** | **80.2** | **80.4** | **78.7** | **78.7** | **65.4** |
| **Brazilian free-tailed bat** | **80.1** | ***81.7*** | **83.5** | **80.6** | **83.6** | **81.5** |  | **81.9** | **80** | **82** | **81.7** | **80.4** | **82.8** | **64.8** |
| **Chinese rufous horseshoe bat** | **80.6** | ***78.8*** | **79.5** | **80** | **84.6** | **80** | **81.9** |  | **81.1** | **93** | **94.2** | **84.7** | **79** | **65.3** |
| **Egyptian rousette** | **79** | ***77.1*** | **79** | **79.1** | **82.9** | **80.2** | **80** | **81.1** |  | **81.4** | **81.6** | **80.4** | **77.2** | **62.3** |
| **Greater Horseshoe bat** | **81.2** | ***78.9*** | **80.7** | **80.2** | **85.5** | **80.2** | **82** | **93** | **81.4** |  | **92.4** | **85** | **79.1** | **64.6** |
| **Pearson's horseshoe bat** | **81.4** | ***78.3*** | **80.2** | **80** | **85** | **80.4** | **81.7** | **94.2** | **81.6** | **92.4** |  | **85.1** | **79.5** | **65.3** |
| **Great roundleaf bat** | **80.5** | ***78.5*** | **80.8** | **78.9** | **84.6** | **78.7** | **80.4** | **84.7** | **80.4** | **85** | **85.1** |  | **79.9** | **64.9** |
| **Little brown bat** | **79.3** | ***78.8*** | **88.3** | **78.3** | **82** | **78.7** | **82.8** | **79** | **77.2** | **79.1** | **79.5** | **79.9** |  | **62.3** |
| **Chicken** | **65.6** | ***63.8*** | **62.9** | **65.4** | **66.5** | **65.4** | **64.8** | **65.3** | **62.3** | **64.6** | **65.3** | **64.9** | **62.3** |  |
| **AVERAGE** | **79.7** | ***78.2*** | **79.6** | **79.5** | **83.0** | **80.0** | **80.3** | **81.7** | **78.5** | **81.8** | **81.9** | **80.1** | **78.8** | **64.5** |


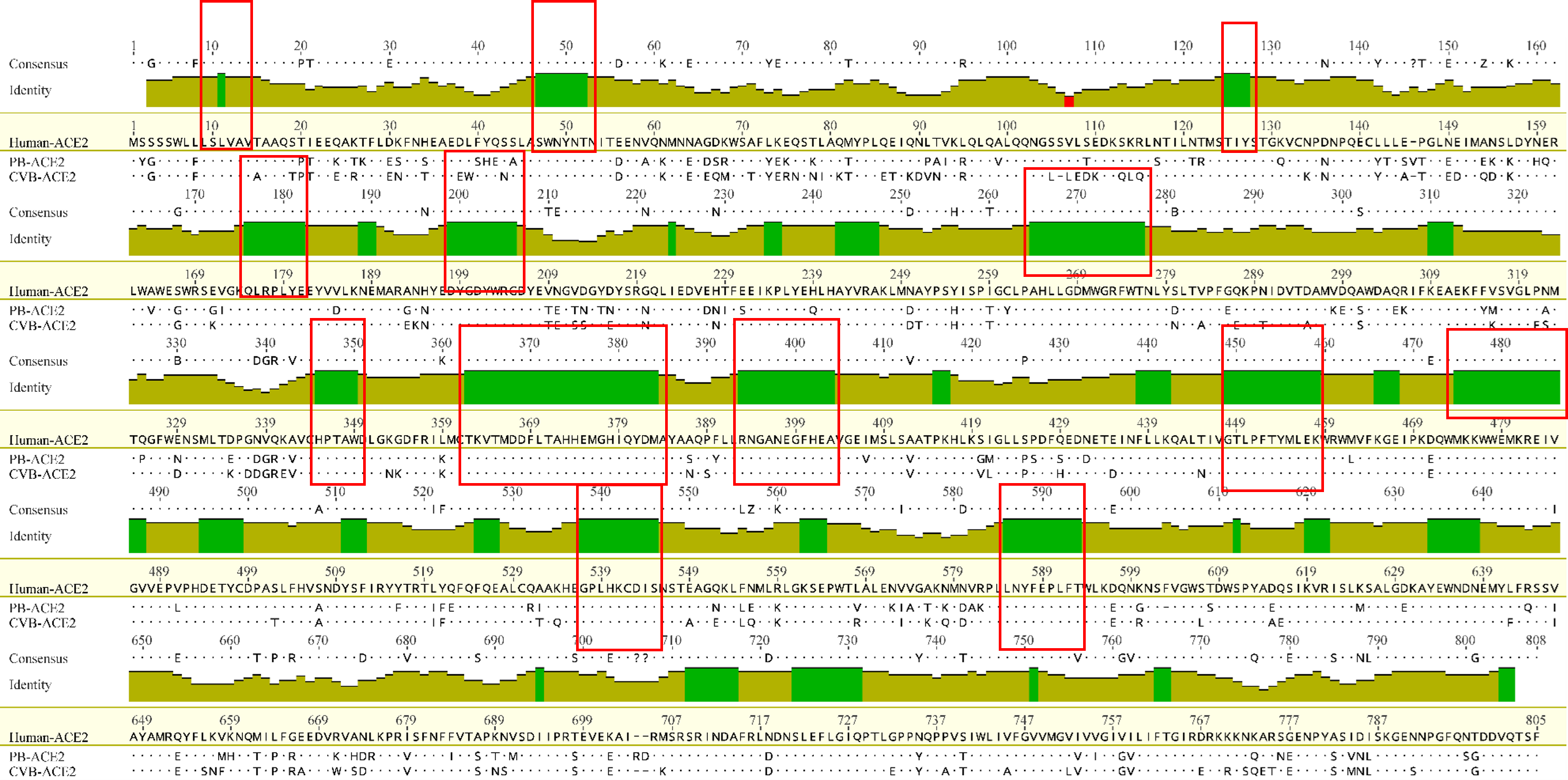


**Figure S1.** Multiple alignment of Human, CVB and PB ACE2 proteins is shown. Regions of 100% identity between the two proteins are shown as green blocks. Residue numbers are indicated on top and both primary amino acid sequence and consensus sequence are shown. Only residues different from human ACE2 are shown.


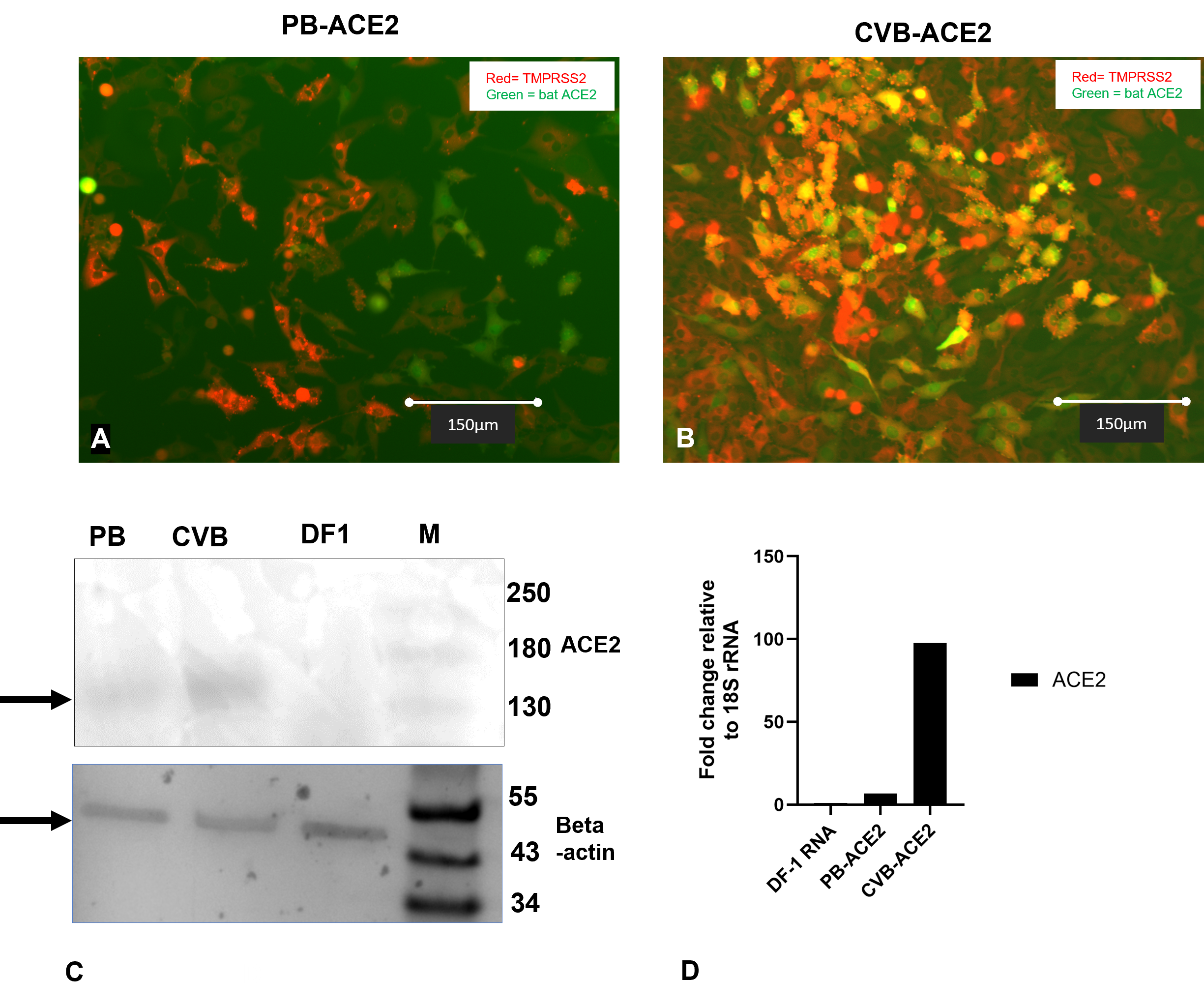


**Figure S2.** Immunofluorescence microscopy of CVB-ACE2 and PB-ACE2 DF-1 cells. Monolayers of PB-ACE2 (**A**) or CVB-ACE2 expressing DF-1 cells (**B**) plated overnight on Greiner µClear flat bottom 96 well plates were imaged with an Invitrogen EVOS M5000 microscope. Scale bar at bottom right represents 20X magnification. Red represents DF-1 cells constitutively expressing human TMPRSS2 with mCherry Red, green represent cells only EGFP expressing CVB-ACE2 (**A**) or PB-ACE2 (**B**) while orange yellow represent cells expressing both hTMPRSS2 and ACE2. Western blot (**C**) demonstrates expression of PB-ACE2 and CVB-ACE2 protein (top panel) and loading control (beta-actin (bottom panel) )as described in materials and methods. Arrows indicate expected size. (**D**) qRT-PCR for fold change in ACE2 transcripts in PB-ACE2 and CVB-ACE2 relative to 18S rRNA is plotted.


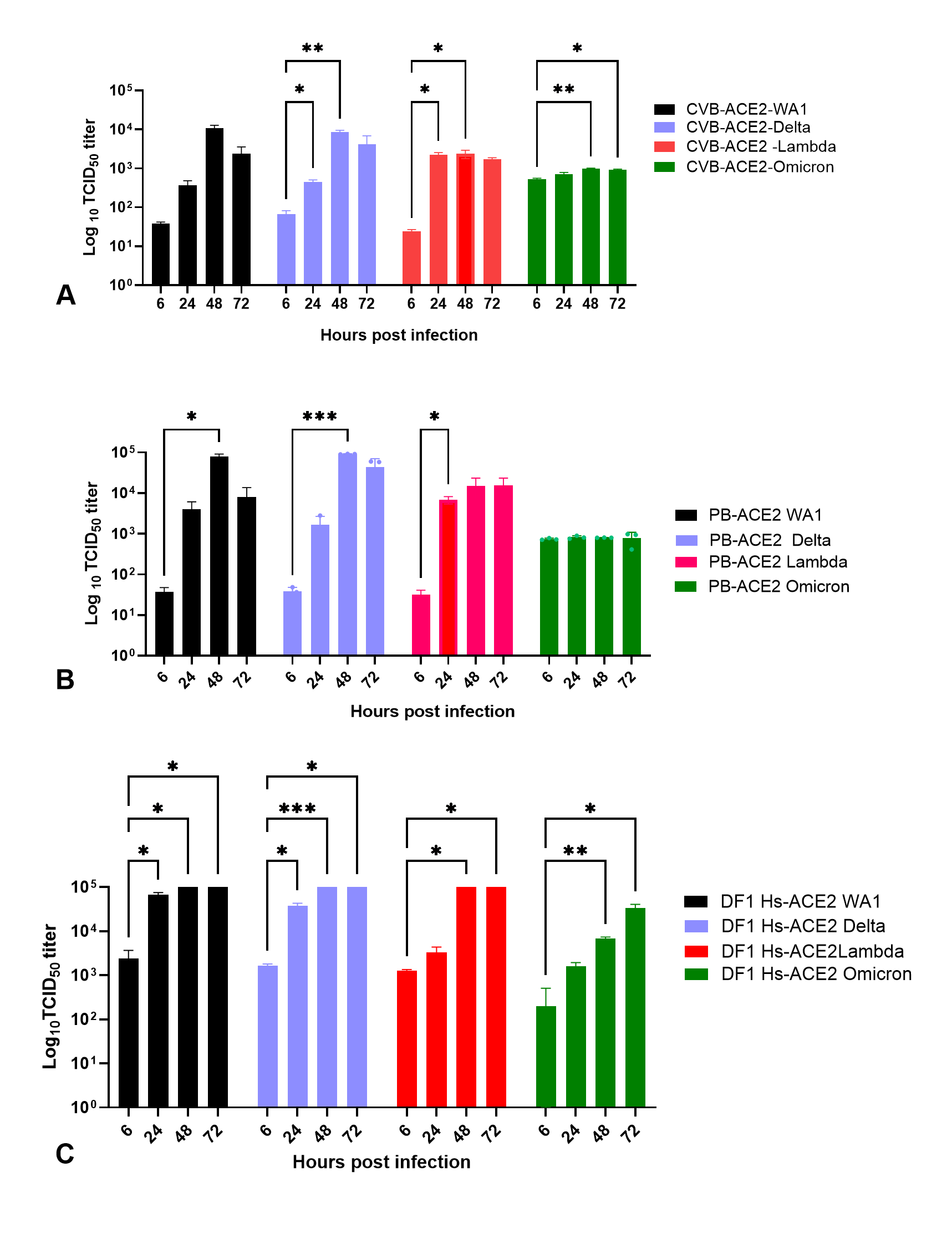


**Figure S3.** Graphs depict log_10_TCID_50_ titers of the SC2 variants used in this study in (**A**) CVB-ACE2 cells, (**B**) PB-ACE2 cells and (**C**) DF-1 cells expressing Hs-ACE2 from 6 h, 24 h, 48 h and 72 h post infection. Error bars represent mean ± SD from three biological replicates. Log_10_TCID_50_ titers are plotted on y-axis against hours post infection on the x-axis. Statistical comparisons were performed with one-way ANOVA using the Geisser-Greenhouse correction and post-hoc Dunnett’s multiple comparison tests and 95% confidence interval and alpha set to 0.05. * = *p* value ≤ 0.05, ** = *p* ≤ 0.005 and *** = *p* ≤ 0.0005.
